# Supplementary material for: Quality-of-life among Syrian refugees residing outside camps in Jordan relative to Jordanians and other countries
Source: PeerJ. 2019 Mar 11;7:e6454. doi: 10.7717/peerj.6454 (PMC6417405; doi:10.7717/peerj.6454)
Supplement: Supplemental Information 1 [file peerj-07-6454-s001.pdf]

```
recode f1 f2 f3 f4 f5 f6 f7 f8 f9 f10 f11 f12 f13 f14 f15 f16 f17 f18 f19
f20 f21 f22 f23 f24 f25 f26 (1=1) (2=2) (3=3) (4=4) (5=5) (ELSE=SYSMIS).

recode f3 f4 f26 (1=5) (2=4) (3=3) (4=2) (5=1).

compute dom1=(mean.6(f3,f4,f10,f15,f16,f17,f18))*4.
compute dom2=(mean.5(f5,f6,f7,f11,f19,f26))*4.
compute dom3=(mean.2(f20,f21,f22))*4.
compute dom4=(mean.6(f8,f9,f12,f13,f14,f23,f24,f25))*4.
compute overall=(mean.2(f1,f2))*4.

compute dom1b=(dom1-4) *(100/16).
compute dom2b=(dom2-4) *(100/16).
compute dom3b=(dom3-4) *(100/16).
compute dom4b=(dom4-4) *(100/16).
compute f1b=(f1-1) *(100/4).
compute f2b=(f2-1) *(100/4).

COUNT TOTAL=F1 TO F26 (1 THRU 5).
SELECT IF (TOTAL>=21).
EXECUTE.

RELIABILITY
  /VARIABLES=f3 f4 f10 f15 f16 f17 f18
  /FORMAT=NOLABELS
  /SCALE(ALPHA)=ALL/MODEL=ALPHA.

RELIABILITY
  /VARIABLES=f5 f6 f7 f11 f26
  /FORMAT=NOLABELS
```

```
  /SCALE(ALPHA)=ALL/MODEL=ALPHA.

RELIABILITY
  /VARIABLES=f20 f21 f22
  /FORMAT=NOLABELS
  /SCALE(ALPHA)=ALL/MODEL=ALPHA.

RELIABILITY
  /VARIABLES=f8 f9 f12 f13 f14 f23 f24 f25
  /FORMAT=NOLABELS
  /SCALE(ALPHA)=ALL/MODEL=ALPHA.
```
