# Supplementary material for: Quality-of-life among Syrian refugees residing outside camps in Jordan relative to Jordanians and other countries
Source: PeerJ. 2019 Mar 11;7:e6454. doi: 10.7717/peerj.6454 (PMC6417405; doi:10.7717/peerj.6454)
Supplement: Supplemental Information 2 [file peerj-07-6454-s002.docx]

| **Variables** | **Syrian Population** | | **ASES Jordanians** | | **LSES Jordanians** | |
| --- | --- | --- | --- | --- | --- | --- |
|  | **Change in Domain Scores** | **P-value** | **Change in Domain Scores** | **P-value** | **Change in Domain Scores** | **P-value** |
| **Physical Health Domain** |  |  |  |  |  |  |
| Intercept | 59.59 | 0.00 | 74.58 | 0.00 | 41.37 | 0.00 |
| Age | -0.18 | 0.00 | -0.14 | 0.01 | --- | --- |
| Chronic Diseases | -2.31 | 0.00 | -5.67 | 0.00 | --- | --- |
| Family size | -0.37 | 0.07 | -0.96 | 0.00 | --- | --- |
| Years of Education | -0.21 | 0.08 | --- | --- | 0.65 | 0.15 |
| Job | 5.09 | 0.00 | 2.78 | 0.03 | --- | --- |
| Marital Status | 1.37 | 0.04 | --- | --- | -2.04 | 0.10 |
| Income | --- | --- | 0.01 | 0.00 | 0.02 | 0.00 |
| Personal description of financial situation | --- | --- | 6.55 | 0.00 | --- | --- |
| **Psychological Health Domain** |  |  |  |  |  |  |
| Intercept | 53.30 | 0.00 | 43.16 | 0.00 | 38.12 | 0.00 |
| Age | -0.10 | 0.03 | --- | --- | --- | --- |
| Chronic Diseases | --- | --- | -2.42 | 0.00 | --- | --- |
| Family size | -0.74 | 0.00 | -0.46 | 0.11 |  | --- |
| Years of Education | --- | --- | 0.39 | 0.02 | 0.94 | 0.04 |
| Job | --- | --- | 3.77 | 0.00 | --- | --- |
| Marital Status |  |  | 2.51 | 0.00 | --- | --- |
| Income | 0.02248 | 0.00 | --- | --- | --- | --- |
| Personal description of financial situation | 2.33516 | 0.12 | 9.35 | 0.00 | 9.76 | 0.00 |
| **Social Relationships Domain** |  |  |  |  |  |  |
| Intercept | 60.50 | 0.00 | 53.63 | 0.01 | 41.18 | 0.00 |
| Age | -0.2029 | 0.00 | --- | --- | --- | --- |
| Chronic Diseases | --- | --- | --- | --- | -5.28 | 0.06 |
| Family size | -0.62 | 0.02 | -0.88 | 0.01 | --- | --- |
| Years of Education | -0.28 | 0.07 | 0.46 | 0.01 | --- | --- |
| Job | --- | --- | --- | --- | --- | --- |
| Marital Status | --- | --- | 1.95 | 0.03 | 4.24 | 0.12 |
| Income | 0.02 | 0.00 | --- | --- | --- | --- |
| Personal description of financial situation | --- | --- | 7.65 | 0.00 | 12.66 | 0.01 |
| **Environmental Health Domain** |  |  |  |  |  |  |
| Intercept | 55.00 | 0.00 | 51.76475 | 0.00 | 20.09 | 0.00 |
| Age | --- | --- | --- | --- | --- | --- |
| Chronic Diseases | -0.72 | 0.09 | --- | --- | 3.43 | 0.05 |
| Family size | --- | --- | -0.91714 | 0.00 | --- | --- |
| Years of Education | -0.35 | 0.00 | --- | --- | 1.23 | 0.03 |
| Job | --- | --- | --- | --- | 5.60 | 0.14 |
| Marital Status | --- | --- | --- | --- | --- | --- |
| Income | 0.01 | 0.04 | 0.01 | 0.00 | --- | --- |
| Gender | -3.25 | 0.00 | -2.43 | 0.09 | --- | --- |
| Personal description of financial situation | --- | --- | 12.19 | 0.00 | 15.77 | 0.00 |
